# The geography and economics of forgoing medical examinations or therapeutic treatments in Italy during the economic crisis

**DOI:** 10.1186/s12889-019-7502-x

**Published:** 2019-09-02

**Authors:** Alessio Petrelli, Aldo Rosano, Alessandra Rossi, Concetta Mirisola, Cesare Cislaghi

**Affiliations:** 1 National Institute for Health, Migration and Poverty (INMP), Rome, Italy; 2 Italian National Institute of Health (ISS), Rome, Italy; 3 Agenzia Nazionale per i Servizi Sanitari Regionali (AGENAS), Rome, Italy

**Keywords:** Unmet needs, Poverty, Socioeconomic, Immigrants, Inequalities, Geographic

## Abstract

**Background:**

In Italy, the number of individuals who have forgone medical examinations or treatments for economic reasons is one of the highest in Europe. During the global economic crisis of 2008, the restrictive policies concerning access to healthcare and the quality of these services, which differs widely throughout the country, may have accentuated the territorial differences in unmet needs, thereby penalizing the more disadvantaged segments of the population.

The study aimed at evaluating the geographical and socioeconomic differences, in particular the risk of poverty, that influence forgoing healthcare services in Italy.

**Methods:**

Cross-sectional Italian data from the 2004–2015 European Survey on Income and Living Conditions (EU-SILC) were used.

Hierarchical logistic models were tested, using as the outcome unmet needs for medical examinations or treatment in the preceding 12 months, and as risk factor the condition of being at risk of poverty. Age, sex, citizenship, educational level, presence of chronic or severely limiting diseases and self-perceived health were used as adjustment factors. Analyses were stratified over three time periods: pre-crisis (2004–2007), initial phase of the crisis (2008–2012) and second phase of the crisis (2013–2015).

**Results:**

In Central Italy and particularly in Southern Italy, a marked increase (9.9% in 2013–2015) was seen in the overall rate of unmet needs as well as in that of unmet needs due to economic reasons. The probability of unmet needs was higher, and increased over time, for those at risk of poverty (aOR = 1.54 in 2004–07, aOR = 1.70 in 2008–12, aOR = 2.21 in 2013–15). Individuals with a low educational level, who had a chronic or severely limiting disease, who perceived their health as not good and immigrants had a higher risk of forgoing healthcare. The regions in Southern Italy had a significantly higher probability of unmet needs.

**Conclusions:**

A strong association was found between the probability of forgoing medical examination or treatment and being at risk of poverty. Study results underline the need for healthcare policies aimed at facilitating access to healthcare services, particularly in the South, by developing a progressive mechanism of contribution to healthcare costs proportional to income and by guaranteeing free access to the poor.

**Electronic supplementary material:**

The online version of this article (10.1186/s12889-019-7502-x) contains supplementary material, which is available to authorized users.

## Background

The effects of the recent global economic crisis on the health of the European population have been the subject of a systematic review that showed a trend of reduced mortality, a deterioration in mental health and an increase in the number of suicides and a worsening in perceived health in Greece and in the Baltic countries [1]. During the crisis the more limited decrease in the number of deaths among the more disadvantaged social groups compared to the general population determined a widening of mortality inequalities [2]; there is also evidence of a widening of inequalities in behavioural risk factors, in particular smoking and alcohol abuse, to the detriment of the more disadvantaged groups [3]. To cope with the effects of the economic recession on their national budgets, many European governments have implemented measures to reduce healthcare spending, as occurred in the UK, Spain and Greece [4], or introduced other forms of co-payment in addition to those already in place for access to services, as happened in Italy [5]. The combined effect of such measures has exposed the most vulnerable segments of the population to greater difficulties in accessing healthcare, resulting in potentially negative effects on health in the medium-long term.

Between 2007 and 2014 in Europe, the prevalence of individuals who declared that they had forgone medical examinations or therapeutic treatment for any reason (from 6.9% in 2007 to 6.7% in 2014) and for economic reasons (from 2.6% in 2007 to 2.4% in 2014) remained constant, despite some differences between countries. Large increases in unmet needs were seen especially in Greece, both for any reason (from 6.7 to 12.7%) and for economic reasons (from 4.6 to 9.7%), but also seen in Belgium and France, though increases were smaller [6]. Further, a study based on the EU-SILC survey conducted in 30 European countries between 2008 and 2013 showed an increase in the number of individuals in the low-income segment of the population who had forgone medical care [7].

Along with Greece, Italy saw the greatest increase in the number of individuals who forwent medical care for economic reasons (from 3.2 to 6.2%), in comparison with a slight increase for any reason (from 6.7 to 7.8%) [6]. Between 2007 and 2013, the number of individuals living in poverty, especially in absolute terms, increased from 4.1 to 7.9%; this increase was even greater in Southern Italy (from 5.8 to 12.6%) [8]. A sharp decrease in employment was also seen in 2008–2016 (− 2.1%), especially among immigrants (− 8.9%) [9].

In Italy, the National Health Service provides universal healthcare, is funded through tax revenues and provides care organized at the regional level. The quality of healthcare is extremely varied throughout the country; the effectiveness of meeting healthcare needs and operative efficiency are worse in the South than in the Centre-North [10, 11]. In the last decade, many factors have contributed to the implementation of restrictive policies concerning access to services that may have introduced potential barriers to universal coverage, thus jeopardizing the equity of access to healthcare. The economic crisis and the often negative budgets of some Italian regions, especially in the South, have in fact forced the Government to impose drastic sovereign debt repayment plans, which have generated an increase in the levels co-payment not associated with the needs of each region. The joint effect of these factors may have contributed to accentuating the differences throughout Italy in unmet healthcare needs and may have penalized the more disadvantaged segments of the population, despite the fact that individuals with low income are exempt from co-payment [12].

The aim of the present study was to evaluate the geographical and socioeconomic differences, especially the risk of poverty, in unmet needs for medical visits and treatment in Italy between 2004 and 2015.

## Methods

This study used the Italian data from the European Union Statistics on Income and Living Conditions (EU-SILC) survey conducted by the Italian National Institute of Statistics (Istat) on a representative sample of the population over the age of 15 residing in Italy from 2004 to 2015 [13].

The survey is conducted annually; since the 2004 edition, it has used a cross-sectional design, adopted in our study, overlaid on a longitudinal survey. The cross-sectional study is based on a two-stage sampling, with stratification of the units of the first stage, i.e. the municipalities stratified by demographic size. The units of the second stage are the families selected from municipal registry offices by means of a systematic choice, with no readmission. All members of families selected are interviewed. The overall sample for the period considered (2004–2015) included 517,143 individuals (*n* = 43,095 yearly average). For the purposes of this study, after having excluded missing values, the analyses were conducted on the not weighted sample of 502,766 individuals [14].

The survey detects numerous variables, which make up the basis of calculation for indicators standardised throughout Europe of the social and economic condition of the population. In particular, information is collected on income, spending, possession of material goods and the quality of life of the households, as well as some information on health, including self-perceived health, chronic conditions, limitations and unmet medical and dental needs [13]. The survey is conducted through interviews at home or by telephone using the Computer Assisted Personal Interview (CAPI) method.

### Statistical analysis

For the purposes of this study we used as indicator of forgone medical examinations or therapeutic treatment the following survey question: “Was there any time during the last 12 months when, in your opinion you really needed a medical examination or treatment for a health problem but you did not receive it?” We considered as a risk factor for forgoing medical examinations or therapeutic treatment the condition of being at risk of poverty, used as an indicator of economic hardship. In accordance with the definition used by Eurostat, the survey considers at risk of poverty those persons who declare an income equal to or below 60% of the median value of the individual distribution of the national equivalised disposable income [15].^1^ To take into account the marked heterogeneity of the distribution of income and of cost of living throughout Italy, we defined risk of poverty as having an income equal to or below 60% of the annual median value in the region of residence. Indeed, if national income were used as the standard for calculating risk of poverty, the results observed would be attributable primarily to the difference in income between the north and south of the country.

As adjustment factors we used age (16–34, 35–49, 50–64, 65–74, 75+), sex (male, female), citizenship (Italian, foreign national), educational level (high, medium, low), presence of chronic or seriously limiting conditions (none, at least one condition) and self-perceived health (good, not good). We stratified the analyses by subdividing the overall period from 2004 to 2015 in 3 phases: pre-crisis 2004–2007, initial phase of the crisis 2008–2012 and second phases of the crisis 2013–2015.

Educational level was based on the International Standard Classification of Education (ISCED): high (upper secondary education, post-secondary non-tertiary education, short cycle tertiary, bachelor or equivalent, master or equivalent, doctorate or equivalent), medium (lower secondary education), low (less than primary education, primary education).

In terms of health, it must be taken into consideration that the probability of forgoing healthcare is a function of the amount of healthcare prescribed, which is strictly correlated to one’s health status. In the analyses, it is thus opportune to take into account the variability in health status of the sample. To this end, we considered the following indicators: chronic or severely limiting conditions and self-perceived health.

The first was created by joining variables concerning the presence of diseases or health problems lasting at least 6 months or that were expected to last at least 6 months and/ or serious limitations in carrying out routine activities lasting at least 6 months.

The second was obtained by grouping the five possible answers to the question “How is your health generally?” in the following two categories: good (very good/ good), not good (not bad/bad/very bad until 2006 and neither good nor bad/bad/very bad as of 2007).

To evaluate the geographical and socioeconomic differences associated with forgoing medical examinations or therapeutic treatment we tested three random intercept hierarchical multivariate logistic regressions models for each time interval, in which the individuals represented the 1st level units and the regions the 2nd level units, adjusting for all the above-mentioned covariates considered at 1st level. Hierarchical models were used because it can be hypothesized that unmet needs have a structure of correlation between individuals that differs between regions of residence both due to the greater homogeneity in the resident population’s socio-demographic characteristics and to the effect of the heterogeneity of the regional healthcare systems in terms of funding and organization. We estimated the geographical differences as regional residual around 1st level intercept, which represents the national mean effect after adjusting for all the covariates considered. The effect of socioeconomic conditions was evaluated through the estimate of the crude (OR) and adjusted (aOR) odds ratios concerning risk of poverty, educational level and citizenship. We also tested the significance of the time trend for these factors over the periods considered. Finally, we tested the interaction between citizenship and education level. In order to evaluate the need to use hierarchical models, we estimated the intraclass correlation coefficient (ICC) with 95% confidence interval (95%CI), a useful measure to test the need for multilevel models. The ICC varies from + 1, when group risks differ but within any group there is no variation, to − 1/(n-1), when group means are equal but the within-group variation is large, where n represents the number of 2nd level statistical unit. We tested the goodness of fit of hierarchical models for each period by comparing crude models (adjusted only by the risk of poverty) with the full models using log-likelihood ratio test. Statistical analyses were performed with STATA.

## Results

**Fig. 1:**
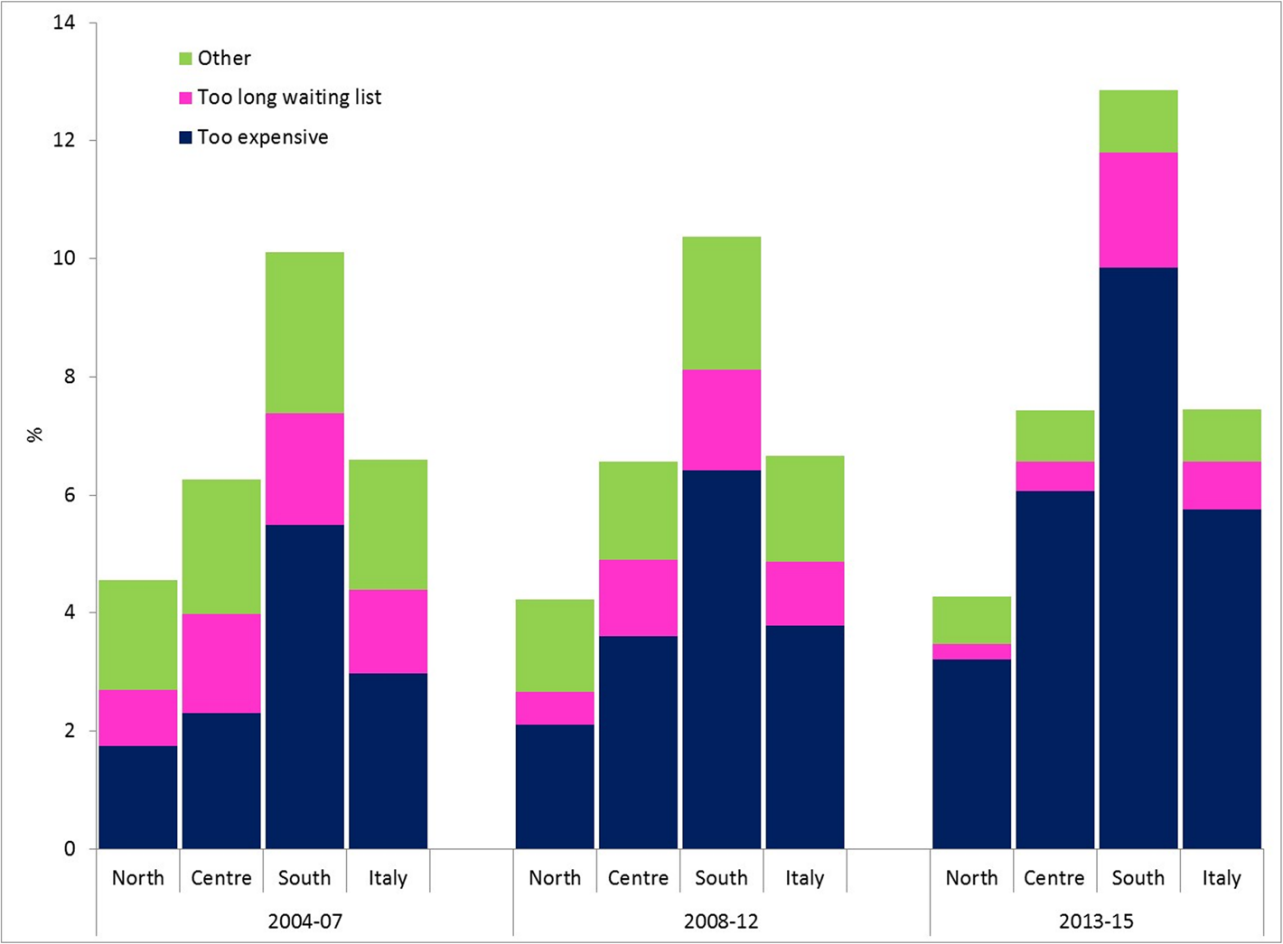
% of forgone specialist medical examinations or treatment in the preceding 12 months per period, geographic area and reason for unmet need

**Fig. 2:**
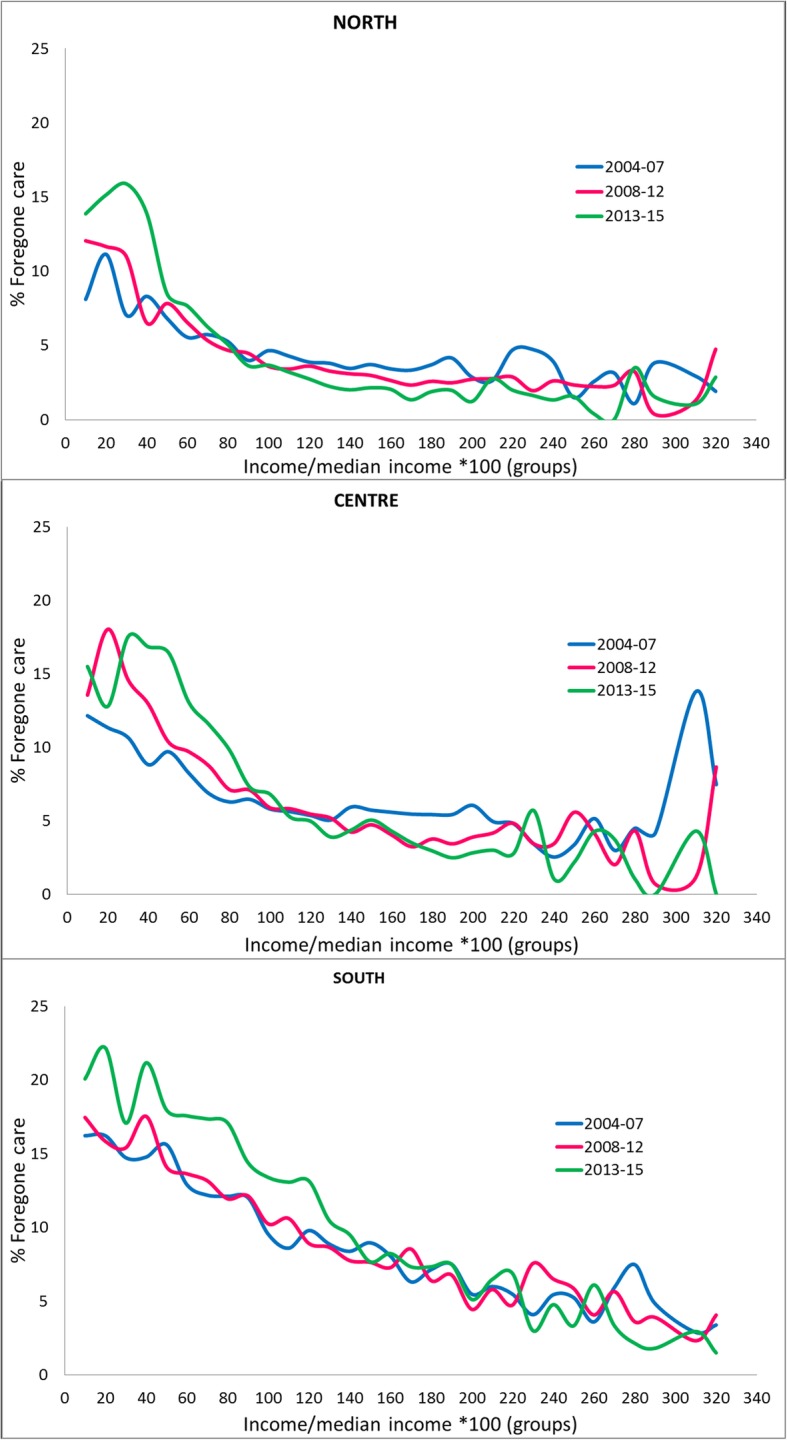
% of unmet needs per income group percentages compared to regional average, period and geographic area

In the time period considered, healthcare forgone for any reason saw a slight increase, from 6.6% between 2004 and 2007 to 6.7% between 2008 and 2012, and to 7.4% between 2013 and 2015. Unmet needs due to economic reasons, however, increased markedly in the same periods (3, 3.8 and 5.8%, respectively), while those due to waiting lists or other reasons decreased. This trend was very heterogenous throughout the country: in the North, overall unmet needs remained stable and markedly lower than the national mean though with an increase for economic reasons, while in the Center and South, an appreciable increase was seen in overall unmet needs as well as in unmet needs due to economic reasons. In absolute terms this was particularly evident in the South (9.9% in the 3-year period 2013–2015) (Fig. 1), especially in Puglia and Calabria (Table 1). Figure 2 shows the distribution of the percentage class of income on median income (regional and annual), in relation to the period and to the geographic area; a general decrease in unmet needs was seen as income increased, for each geographic area and in all time periods. In the last 3-year period (2013–2015), the percentage of forgone care was higher only for lower incomes, and consistently lower for higher incomes than in preceding periods, heterogeneously by geographic area. In fact, the income threshold for which unmet needs was higher in preceding periods than in the last 3-year period was lower in the North (80% of regional median income) than in the Center (110% of regional median income) and above all than in the South (150% of regional median income).

**Table 1:** % of forgone medical visits or specialist treatment in the preceding 12 months for any reason per period and sample characteristics

		2004–2007							2008–2012							2013–2015						
		No		Yes		Total		*p*	No		Yes		Total		*p*	No		Yes		Total		*p*
		n	%	n	%	n	%		n	%	n	%	n	%		n	%	n	%	n	%	
	Total	177,174	93.4	12,519	6.6	189,693	100		189,373	93.3	13,521	6.7	202,894	100		101,977	92.6	8202	7.4	110,179	100	
Sex	Males	85,677	94.2	5308	5.8	90,985	48.0	<.0001	91,562	94.3	5576	5.7	97,138	47.9	<.0001	49,120	93.5	3438	6.5	52,558	47.7	<.0001
	Females	91,497	92.7	7211	7.3	98,708	52.0		97,811	92.5	7945	7.5	105,756	52.1		52,857	91.7	4764	8.3	57,621	52.3	
Age class	16–34	48,136	95.6	2231	4.4	50,367	26.6	<.0001	45,652	95.6	2113	4.4	47,765	23.5	<.0001	21,565	96.1	881	3.9	22,446	20.4	<.0001
	35–49	47,448	92.9	3628	7.1	51,076	26.9		51,558	92.8	4013	7.2	55,571	27.4		26,783	92.3	2246	7.7	29,029	26.3	
	50–64	40,375	92.5	3284	7.5	43,659	23.0		44,870	92.5	3625	7.5	48,495	23.9		25,927	91.4	2450	8.6	28,377	25.8	
	65–74	22,389	92.6	1784	7.4	24,173	12.7		25,159	92.7	1975	7.3	27,134	13.4		14,733	91.6	1346	8.4	16,079	14.6	
	75+	18,826	92.2	1592	7.8	20,418	10.8		22,134	92.5	1795	7.5	23,929	11.8		12,969	91.0	1279	9.0	14,248	12.9	
Risk of poverty	No	150,100	94.0	9569	6.0	159,669	84.2	<.0001	160,116	94.2	9897	5.8	170,013	83.8	<.0001	85,744	93.9	5531	6.1	91,275	82.8	<.0001
	Yes	27,074	90.2	2950	9.8	30,024	15.8		29,257	89.0	3624	11.0	32,881	16.2		16,233	85.9	2671	14.1	18,904	17.2	
Educational level	High	79,545	94.8	4367	5.2	83,912	44.2	<.0001	95,538	95.0	4985	5.0	100,523	49.5	<.0001	58,000	95.1	2999	4.9	60,999	55.4	<.0001
	Medium	50,005	93.4	3528	6.6	53,533	28.2		52,906	92.4	4375	7.6	57,281	28.2		26,756	90.5	2807	9.5	29,563	26.8	
	Low	47,624	91.1	4624	8.9	52,248	27.5		40,929	90.8	4161	9.2	45,090	22.2		17,221	87.8	2396	12.2	19,617	17.8	
Citizenship	Italian	172,600	93.4	12,134	6.6	184,734	97.4	< 0.005	182,215	93.4	12,773	6.6	194,988	96.1	<.0001	97,657	92.8	7600	7.2	105,257	95.5	<.0001
	Foreign national	4574	92.2	385	7.8	4959	2.6		7158	90.5	748	9.5	7906	3.9		4320	87.8	602	12.2	4922	4.5	
Chronic disease or severe limitations	No	140,617	94.9	7503	5.1	148,120	78.1	<.0001	147,165	94.9	7836	5.1	155,001	76.4	<.0001	77,439	95.0	4067	5.0	81,506	74.0	<.0001
	Yes	36,557	87.9	5016	12.1	41,573	21.9		42,208	88.1	5685	11.9	47,893	23.6		24,538	85.6	4135	14.4	28,673	26.0	
Self-perceived health	Good	108,753	96.1	4391	3.9	113,144	59.6	<.0001	129,133	96.0	5400	4.0	134,533	66.3	<.0001	71,429	95.8	3143	4.2	74,572	67.7	<.0001
	Not good	68,421	89.4	8128	10.6	76,549	40.4		60,240	88.1	8121	11.9	68,361	33.7		30,548	85.8	5059	14.2	35,607	32.3	
Region	Piemonte	11,314	94.7	638	5.3	11,952	6.3	<.0001	11,985	95.1	616	4.9	12,601	6.2	<.0001	7427	96.6	264	3.4	7691	7.0	<.0001
	Valle d’Aosta	3010	96.4	111	3.6	3121	1.6		3194	97.3	90	2.7	3284	1.6		1889	95.1	97	4.9	1986	1.8	
	Lombardia	19,742	95.6	910	4.4	20,652	10.9		20,016	95.7	889	4.3	20,905	10.3		10,516	95.4	512	4.6	11,028	10.0	
	Bolzano	4241	96.2	168	3.8	4409	2.3		3892	96.7	133	3.3	4025	2.0		1214	97.4	32	2.6	1246	1.1	
	Trento	3305	97.0	103	3.0	3408	1.8		3583	97.7	86	2.3	3669	1.8		2437	98.9	28	1.1	2465	2.2	
	Veneto	14,523	95.1	751	4.9	15,274	8.1		15,023	96.3	576	3.7	15,599	7.7		8380	96.8	280	3.2	8660	7.9	
	Friuli-Venezia Giulia	7475	96.4	280	3.6	7755	4.1		8772	95.8	381	4.2	9153	4.5		5842	96.0	244	4.0	6086	5.5	
	Liguria	7201	95.5	341	4.5	7542	4.0		8249	95.4	399	4.6	8648	4.3		5451	94.8	296	5.2	5747	5.2	
	Emilia-Romagna	13,232	95.0	703	5.0	13,935	7.3		13,397	94.9	716	5.1	14,113	7.0		7615	93.7	513	6.3	8128	7.4	
	Toscana	12,836	94.0	825	6.0	13,661	7.2		12,771	93.8	839	6.2	13,610	6.7		6417	96.6	229	3.4	6646	6.0	
	Umbria	8293	95.9	359	4.1	8652	4.6		8782	95.2	441	4.8	9223	4.5		3772	92.8	292	7.2	4064	3.7	
	Marche	9124	93.8	602	6.2	9726	5.1		9513	93.2	699	6.8	10,212	5.0		6049	90.2	660	9.8	6709	6.1	
	Lazio	12,556	92.1	1071	7.9	13,627	7.2		14,555	92.3	1222	7.7	15,777	7.8		7830	91.2	753	8.8	8583	7.8	
	Abruzzo	4607	93.0	348	7.0	4955	2.6		4171	90.6	434	9.4	4605	2.3		2363	90.5	249	9.5	2612	2.4	
	Molise	3373	91.5	312	8.5	3685	1.9		3734	92.8	289	7.2	4023	2.0		1755	91.3	167	8.7	1922	1.7	
	Campania	11,323	90.3	1212	9.7	12,535	6.6		12,818	90.9	1279	9.1	14,097	6.9		5787	87.0	867	13.0	6654	6.0	
	Puglia	8134	88.0	1104	12.0	9238	4.9		9036	86.3	1436	13.7	10,472	5.2		4146	80.6	1001	19.4	5147	4.7	
	Basilicata	3862	89.4	457	10.6	4319	2.3		4329	90.9	432	9.1	4761	2.3		2029	93.4	143	6.6	2172	2.0	
	Calabria	4825	87.8	669	12.2	5494	2.9		5841	87.7	817	12.3	6658	3.3		3340	83.1	681	16.9	4021	3.6	
	Sicilia	8828	90.1	972	9.9	9800	5.2		10,314	89.3	1230	10.7	11,544	5.7		5308	90.5	560	9.5	5868	5.3	
	Sardegna	5370	90.2	583	9.8	5953	3.1		5398	91.3	517	8.7	5915	2.9		2410	87.8	334	12.2	2744	2.5

In the three time periods considered, 15.8%, 16.2% and 17.2% of the sample, respectively, was at risk of poverty. The prevalence of forgoing medical examinations or treatment was significantly higher, and with an increasing trend, among those at risk of poverty compared to those who were not (9.8% vs 6.0% between 2004 and 2007, 11.0% vs 5.8% between 2008 and 2012 and 14.1% vs 6.1% between 2013 and 2015).

The percentage of persons who had unmet needs increased with age, with the exception of the age class 65–74 years. Further, the percentage of unmet needs was significantly higher, and increased over time, for those with a low or medium educational level, as well as for foreign nationals (Table 1).

Table 2 shows the adjusted odds ratios (aOR) of the hierarchical logistic models stratified for the three time periods, in which the association between factors considered and forgoing medical examinations or treatment in the preceding 12 months was evaluated (see also Additional file 1: Table S1 showing crude and adjusted odds ratios).

**Table 2:** Adjusted Odds Ratios (aOR) and 95%CI for factors associated with forgone medical visits or specialist treatment in the preceding 12 months for any reason. Hierarchical logistic models per period

	2004–2007				2008–2012				2013–2015			
	aOR	95%CI		*p*-value	aOR	95%CI		*p*-value	aOR	95%CI		*p*-value
Sex												
Males	1	–	–	<.0001	1	–	–	<.0001	1	–	–	<.0001
Females	1.23	1.19	1.28		1.26	1.21	1.30		1.16	1.10	1.22	
Age class												
16–34	1	–	–	<.0001	1	–	–	<.0001	1	–	–	<.0001
35–49	1.27	1.20	1.35		1.38	1.30	1.46		1.74	1.59	1.89	
50–64	1.04	0.97	1.10		1.07	1.01	1.14		1.43	1.31	1.56	
65–74	0.79	0.73	0.86		0.78	0.73	0.85		1.01	0.91	1.12	
75+	0.69	0.64	0.75		0.61	0.56	0.66		0.71	0.63	0.79	
Risk of poverty*												
No	1	–	–	<.0001	1	–	–	<.0001	1	–	–	<.0001
Yes	1.54	1.47	1.61		1.70	1.63	1.78		2.21	2.09	2.34	
Educational level*												
High	1	–	–	<.0001	1	–	–	<.0001	1	–	–	<.0001
Medium	1.10	1.05	1.16		1.29	1.23	1.35		1.53	1.45	1.63	
Low	1.19	1.13	1.26		1.29	1.22	1.36		1.62	1.51	1.75	
Citizenship**												
Italian	1	–	–	<.0001	1	–	–	<.0001	1	–	–	<.0001
Foreign national	1.61	1.44	1.80		1.85	1.71	2.01		2.19	1.98	2.42	
Chronic disease or severe limitations												
No	1	–	–	<.0001	1	–	–	<.0001	1	–	–	<.0001
Yes	1.87	1.79	1.95		1.75	1.67	1.82		2.12	1.99	2.25	
Self-perceived health												
Good	1	–	–	<.0001	1	–	–	<.0001	1	–	–	<.0001
Not good	2.55	2.43	2.67		2.85	2.72	2.99		2.66	2.50	2.84	
Region												
Piemonte	1	–	–	<.0001	1	–	–	<.0001	1	–	–	<.0001
Valle d’Aosta	0.64	0.52	0.78		0.58	0.46	0.72		1.38	1.08	1.76	
Liguria	0.85	0.74	0.98		0.98	0.86	1.12		1.54	1.29	1.83	
Lombardia	0.80	0.72	0.89		0.88	0.79	0.98		1.46	1.25	1.71	
Bolzano	0.79	0.66	0.94		0.74	0.61	0.90		0.87	0.60	1.27	
Trento	0.59	0.47	0.73		0.53	0.42	0.67		0.38	0.26	0.56	
Veneto	0.87	0.78	0.98		0.76	0.68	0.86		1.07	0.89	1.27	
Friuli-Venezia Giulia	0.64	0.55	0.74		0.84	0.74	0.96		1.40	1.17	1.68	
Emilia-Romagna	0.89	0.80	0.99		1.03	0.92	1.15		1.95	1.67	2.28	
Toscana	1.16	1.04	1.29		1.35	1.21	1.51		1.18	0.98	1.41	
Umbria	0.75	0.66	0.86		1.03	0.91	1.18		2.54	2.13	3.03	
Marche	1.15	1.03	1.29		1.43	1.28	1.60		3.46	2.97	4.02	
Lazio	1.51	1.36	1.67		1.72	1.56	1.91		3.22	2.78	3.73	
Abruzzo	1.37	1.19	1.57		2.20	1.93	2.50		3.43	2.85	4.13	
Molise	1.62	1.40	1.87		1.54	1.33	1.78		3.35	2.72	4.12	
Campania	2.08	1.88	2.31		2.23	2.02	2.47		5.24	4.52	6.07	
Puglia	2.58	2.33	2.87		3.39	3.07	3.75		8.21	7.09	9.50	
Basilicata	2.14	1.88	2.43		2.04	1.79	2.32		2.35	1.89	2.91	
Calabria	2.37	2.11	2.67		2.64	2.36	2.95		5.51	4.73	6.43	
Sicilia	1.97	1.77	2.18		2.42	2.19	2.69		3.14	2.69	3.67	
Sardegna	1.90	1.69	2.14		1.86	1.65	2.11		4.38	3.67	5.21	
Educational attainment level by citizenship												
Medium vs High among Italians	1.11	1.06	1.17	< 0.01	1.31	1.26	1.38	< 0.01	1.59	1.49	1.69	<.0001
Low vs High among Italians	1.21	1.15	1.28		1.32	1.24	1.39		1.68	1.55	1.81	
Medium vs High among foreign national	0.87	0.68	1.12		0.97	0.82	1.16		1.06	0.86	1.31	
Low vs High among foreign national	0.73	0.54	0.99		0.96	0.74	1.23		1.02	0.73	1.43	
ICC (95%CI)	0.063 (0.034–0.106)				0.072 (0.040–0.126)				0.132 (0.076–0.221)			
Likelihood Ratio Test for goodness of fit	< 0.0001				< 0.0001				< 0.0001			

*p*-value trend: * < 0.05, ** < 0.01

A higher odd of unmet needs can be observed for those at risk of poverty, with an increasing trend over the three time periods (aOR = 1.54 in 2004–07, aOR = 1.70 in 2008–12, aOR = 2.21 in 2013–15). Women had a greater probability of forgoing medical care in all time periods (aOR = 1.23 in 2004–07, aOR = 1.26 in 2008–12, aOR = 1.16 in 2013–15).

The aOR were highest in the age class 35–49 years, then decreased to the point of being below 1 in the oldest age class (75+). This trend was observed in all time periods, with increasing intensity. In addition, a positive association was seen among those who had a medium educational level; this association increased over the three periods (aOR = 1.10 in 2004–07, aOR = 1.29 in 2008–12, aOR = 1.53 in 2013–15). A positive association was also seen for those with a low educational level (aOR = 1.19 in 2004–07, aOR = 1.29 in 2008–12, aOR = 1.62 in 2013–15), for foreign nationals (aOR = 1.61 in 2004–07, aOR = 1.85 in 2008–12, aOR = 2.19 in 2013–15), for those with chronic or severely limiting conditions (aOR = 1.87 in 2004–07, aOR = 1.75 in 2008–12, aOR = 2.12 in 2013–15) and for those who perceived their health as not good (aOR = 2.55 in 2004–07, aOR = 2.85 in 2008–12, aOR = 2.66 in 2013–15). The interaction between education level and citizenship resulted statistically significant: the association between education level and forgone care is present among Italians but not among foreign nationals. The ICC (95%IC) was 0.063 (0.034–0.106) in the first period, 0.072 (0.040–0.126) in the second and 0.132 (0.076–0.221) in the third. Both extremes of the confidence intervals was greater than 0, so that hierarchical models were good enough to account for the clustering effect when analyzing data.

**Fig. 3:**
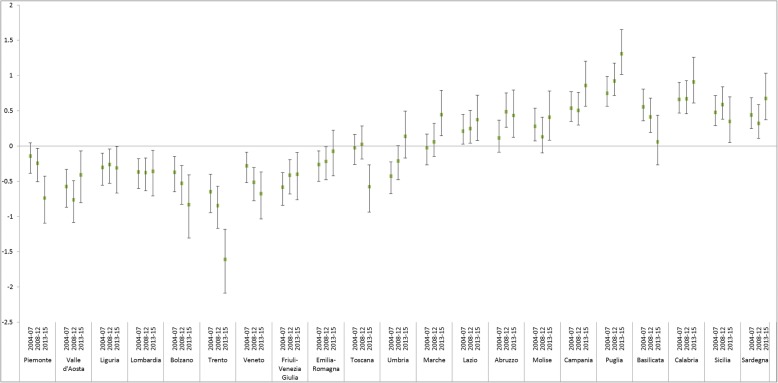
2nd level residuals of hierarchical logistic models

Figure 3 shows the 2nd residuals (regions) around the intercept of the hierarchical logistic models for the three time periods examined. For each region, three lines representing the deviation from the null value for each period are drawn. This could be interpreted as the national average of forgone care, after adjustment for all the covariates included in the models. Considerable national heterogeneity can be seen, with values for unmet needs worsening as one moves further South and with geographic heterogeneity increasing over time. In particular, all factors being equal, unmet needs in the regions in northern Italy were significantly fewer than the national average, with the exception of Piemonte in 2004–2007 and Emilia-Romagna in 2013–2015. The unmet needs in the southern regions were significantly higher than the national average, except in Abruzzo and Sardegna between 2004 and 2007, in Molise between 2008 and 2012 and in Basilicata between 2013 and 2015. All the regions in central Italy except Toscana worsened over time, Marche and Lazio significantly so in the last time period.

## Discussion

Our study highlights a strong association between the forgoing medical examinations or treatment and the condition of being at risk of poverty; this association has become stronger over time. The probability of having unmet needs is greater in women, foreign nationals and those who have a low or medium educational level.

These results are in accordance with the hypothesis that the global economic crisis has played a role in accentuating socioeconomic differences in accessing healthcare. Additional analyses performed in this study show an increase in forgoing medical examinations for economic reasons in the period examined especially in the poorer segments of the population: the percentage of unmet needs increased from 10.8% in the period prior to the economic crisis to 15.4% in the second phase of the crisis for those persons in the first income quintile, while it decreased from 4.2 to 2.4% for the wealthier.

We also observed strong territorial imbalances, with the southern regions at a disadvantage and those in central Italy only in the last time period. These results are concerning, given that such a national distribution of health inequities reflects the health status of the population. Indeed, the life expectancy at birth of individuals residing in southern Italy is 1 year lower than it is for those living in the North (82 years vs 83.1 years) [16], and the percentage of persons who declare their health as bad or very bad is strikingly higher in the South (9.8% vs 5.8% in the North) [17]. These imbalances are even more serious due to the fact that the proportion of the population in the South with an increase in unmet needs over the last 3 years in relation to income is much higher than it is in either the North or the Center, and it involves ever larger groups of the population at risk of poverty.

This situation should give rise to policies aimed at facilitating access to healthcare, particularly in the South, by limiting the need to pay out of pocket. This could be achieved by reducing co-payment, and even extending the social categories that are exempt, and by improving the efficiency of public healthcare facilities so as to reduce the need to resort to private services. Current policies, however, go in the opposite direction; the regions with the highest deficit, in particular those of southern Italy, have been subject to greater co-participation in healthcare spending since 2011 in an attempt to consolidate regional budgets [18]. An effect deriving from this situation has been a growth in the private healthcare sector, which is often able to charge fees that are lower than those in co-payment in public facilities thanks to the joint effect of the development of health technologies, which has reduced the average cost of specialist care, and the fact that fees at public facilities have not been revised for many years and may thus be inflated [19]. This phenomenon is particularly accentuated in the South and is in contrast with the needed reduction in co-participation in healthcare spending, as the reduction in public healthcare services results in a further deficit in the entire healthcare sector. The phenomenon concerns both outpatient diagnostics and treatment as the care setting of many services has been transferred from the hospital to outpatient clinics. This may also explain why the wealthier segment of the population has fewer unmet needs [20].

Our study demonstrates that even the less educated and foreign nationals have a greater likelihood of unmet needs, and these inequalities seem to have increased over time. Moreover, the condition of being an immigrant is an independent predictor of unmet needs, regardless of education level. A previous study highlighted the fact that foreign nationals with a valid residence permit have a higher risk of unmet medical needs than do Italian citizens [21]. Although the Italian healthcare system provides universal coverage, language, cultural and administrative difficulties may be a barrier to accessing healthcare [22].

Throughout Europe, the policies implemented to contain healthcare spending to offset the effects of the economic crisis have impeded access to healthcare. In many countries besides Italy, for example in the Czech Republic, France, Latvia, Romania and Spain, co-payment has been reinforced despite clear evidence supporting the fact that while co-payment may lead to a reduction in the consumption of inappropriate care, it may also result in a reduction in appropriate and essential care, further penalizing the more socially vulnerable population. The differences in healthcare access seem more emphasized during the economic crisis, along with the increase in unemployment and the reduction in disposable income, to the detriment of the more disadvantaged in the population. In the UK, Greece and Spain, where policy makers have implemented measures to reduce healthcare spending, the effects on health have been negative [23]. In addition, the austerity regime in Greece determined by drastic policies to contain public spending has contributed to increasing forgone healthcare due to economic reasons, especially among the poor, people with lower income and the unemployed [24, 25].

Instead, a decrease in unmet needs has been seen in those countries that have reduced spending co-participation, as Croatia did in 2011.

In addition to unmet needs among the poorer segments of the population, there have been more unmet needs in those countries with greater economic inequalities. During the recession, countries with a more equitable distribution of income were able to contrast the reduction in healthcare access more effectively, particularly for the more disadvantaged groups [7, 26]. Income support measures for the unemployed and those not in the labour force or social welfare for poorer families have proven to be effective in offsetting the impact of the crisis on inequities in healthcare access [4, 27].

### Strengths and limitations

The study aimed to analyse the geographical imbalances in Italy and the socioeconomic effects of unmet needs during the global economic crisis. The analyses were performed on a very large sample which represented the resident population in Italy and which had extensive temporal and spatial components. Although the study was conducted using data from a cross-sectional survey, we believe that the risk of bias due to the possibility of an inverse relationship between unmet needs and risk of poverty is quite limited as it is difficult to imagine that a considerable amount of poverty can have been generated by healthcare spending in the Italian healthcare system, which is based on universal access. Furthermore, the reason for forgoing care is subjective, and it is worth underlining that the increase in unmet needs for economic reasons was accompanied by a decrease in unmet needs attributable to other factors, suggesting that at least some of that increase can be explained by a variation in the perception of the motivations for forgoing care, which in a period of economic crisis can more easily be attributed to economic reasons.

Nevertheless, some factors suggest that some results presented in this study should be considered with more caution. First of all, unmet needs, as detected by the EU-SILC survey, is a subjective concept. It is measured by asking interviewees whether they had not been able to receive medical care or treatment in the preceding 12 months despite needing it. The perception of need is strictly tied to the subjective representation of illness, as are the expectations held towards the healthcare service, factors which can vary from one country to another [28] as well as within a country, and can vary between different social classes. This may introduce a distortion in the estimates of risk of unmet needs, the direction of which is difficult to hypothesize. Another limitation of the study is due to the small number of level 2 units in the multilevel models, which means that an accurate estimation of the standard error cannot be guaranteed [29]. However, studies conducted using EU-SILC data are often based on multilevel models, usually considering a limited number of level 2 units. Reliable results were obtained when sensitivity analyses were conducted comparing multilevel models with more traditional approaches [30, 31]. In any case, we cannot rule out the possibility of a slight inaccuracy in estimating standard errors for regional effect. Finally, it must be taken into consideration that as the overall number of healthcare services provided by public and private facilities is increasing, as evidenced by numerous sources, it is natural to hypothesize that the number of individuals who will forgo care will tend to increase. It would thus be important to estimate how many services are forgone, along with the percentage of individuals who have forgone one or more services. Currently, however, no information system or national sample survey allows this type of analysis.

## Conclusions

Overall, the Italian National Health Service seems to have successfully managed the impact of the crisis, demonstrating great resilience to the potential barriers to access resulting from the policies to contain healthcare spending. Nevertheless, the territorial and social imbalances in terms of unmet needs confirm that equitable healthcare access must remain a priority for public health policy.

Healthcare policies must first of all guarantee the entire population, and especially the more vulnerable, equal opportunity of access to healthcare facilities and the meeting of their medical needs by means of progressive measures. Of particular importance is the need to revise the system of contributing to healthcare spending so that it takes into greater account the economic status of each individual so as to reduce the need to access private services, eliminating above all the threshold effect of the current exemption system, which determines the loss of exemption in a dichotomous manner. At the same time developing a progressive mechanism proportional to income must be developed that in any case guarantees free access to the poor.

The recent introduction of an important measure to contrast poverty and social exclusion must be mentioned, the so-called inclusion income, which foresees both progressive economic benefits in relation to family income and specific social inclusion projects aimed at supporting employment search, as well as social-health services pathways that foster the independence of the family [32].

Finally, the relevance of these data in support of developing the criteria for funding regional health services must be mentioned so as to reduce the imbalances in the mechanism of allocating healthcare funding, which takes into only partial account the differences in needs of the various segments of the population, having factored in the variability in efficiency of providing healthcare services. The value of the Italian universal healthcare system appears to be in contrast with the differences among the social classes and between the regions of Italy highlighted in this study in terms of unmet needs.

However, a better reproportioning of funding is not enough if the efficiency of the system remains as imbalanced between the regions as it is. The same per capita resources, adjusted for the volume of needs, can lead to far more services in some efficient regions, thereby reducing waiting times. Alongside this there are problems concerning the appropriateness of prescribed care; this mechanism should consider needs objectively, thereby reducing the differences in demand based on anxiety or lack of a proper health literacy.

## Additional file

Additional file 1: **Table S1.** Crude (OR) and adjusted Odds Ratios (aOR) with 95%CI and *p*-value for factors associated with forgone medical visits or specialist treatment in the preceding 12 months for any reason. Hierarchical logistic models per period. (XLSX 25 kb)

## Data Availability

The data that support the findings of this study are available from Istat but restrictions apply to the availability of these data, which were used under license for the current study, and so are not publicly available. Data are however available from the authors upon reasonable request and with permission of ISTAT.

## Abbreviations

CAPI: Computer Assisted Personal Interview
EU-SILC: European Union Statistics on Income and Living Conditions
ICC: Intraclass Correlation Coefficient
ISCED: International Standard Classification of Education
Istat: National Institute of Statistics
OR: Odds Ratio

## Footnotes

1 Equivalised income is used to compare the income of households with a number of members and is calculated by dividing the value of household income by an opportune correction coefficient (equivalence scales) that takes into account the effect of economy of scale in household spending. The equivalence scale used throughout Europe per the EU-SILC survey attributes a value of 1 to the first adult, 0.5 to every other adult present in the household and 0.3 to each member below the age of 14.
